# Machine-Learning-Assisted Construction of Ternary Convex Hull Diagrams

**DOI:** 10.1021/acs.jcim.3c01391

**Published:** 2024-01-25

**Authors:** Hugo Rossignol, Michail Minotakis, Matteo Cobelli, Stefano Sanvito

**Affiliations:** School of Physics and CRANN Institute, Trinity College Dublin, College Green, Dublin 2, Ireland

## Abstract

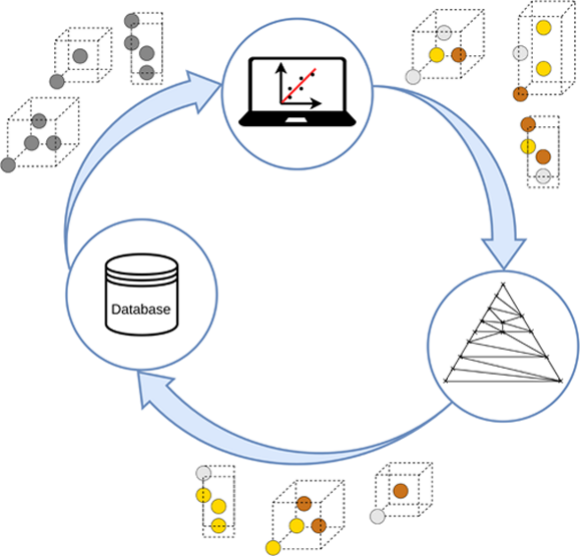

In the search for novel intermetallic ternary alloys, much of the effort goes into performing a large number of *ab initio* calculations covering a wide range of compositions and structures. These are essential to building a reliable convex hull diagram. While density functional theory (DFT) provides accurate predictions for many systems, its computational overheads set a throughput limit on the number of hypothetical phases that can be probed. Here, we demonstrate how an ensemble of machine-learning (ML) spectral neighbor-analysis potentials (SNAPs) can be integrated into a workflow for the construction of accurate ternary convex hull diagrams, highlighting regions that are fertile for materials discovery. Our workflow relies on using available binary-alloy data both to train the SNAP models and to create prototypes for ternary phases. From the prototype structures, all unique ternary decorations are created and used to form a pool of candidate compounds. The SNAPs ensemble is then used to prerelax the structures and screen the most favorable prototypes before using DFT to build the final phase diagram. As constructed, the proposed workflow relies on no extra first-principles data to train the ML surrogate model and yields a DFT-level accurate convex hull. We demonstrate its efficacy by investigating the Cu–Ag–Au and Mo–Ta–W ternary systems.

## 1 Introduction

Systematic materials design aims to develop methods that can help accelerate the discovery of compounds with tailor-made properties, fit for certain applications. The large investment in the area, not least through the materials genome initiative,^1^ underpins the importance of searching for novel compounds to bolster technological progress. Atomistic simulations provide a suitable pathway to achieve this goal, since the search can be performed systematically, at low cost, and with complete control over structure and composition. Density functional theory (DFT) calculations are notably used to predict material properties *in silico*, such as material stability or elastic responses. By performing property predictions across a large range of prototype structures in the form of high-throughput studies,^2^ novel magnetic,^3^ high hardness,^4^ and battery materials^5^ have been discovered. Extensive databases, grouping large numbers of such calculations, have been created and are open to the community. These include AFLOWlib,^6^ Materials Project,^7^ OQMD,^8^ and NOMAD.^9^ While such studies remain faster than experimental investigations, the compositional and structural spaces to be searched are incredibly large, limiting the scope of the application of pure DFT workflows. Importantly, such a limitation in sampling capacity becomes increasingly critical as the number of elements per compound grows, despite the anticipation that a majority of future compound discoveries will be highly multielemental.^10^ In order to address this issue and harness the data available from existing *ab initio* calculations, machine learning (ML) has proven to be a very powerful tool as it typically comes at a fraction of the DFT computational cost.

The first step in high-throughput computational studies consists of identifying stable compounds by finding a stoichiometry and an associated structure that can be formed. In order to assess the stability of a given structure, the appropriate convex hull diagram must be calculated. The proximity between a compound’s enthalpy of formation, Δ*H*_f_, and the closest tie-line on the convex hull serves as a criterion for evaluating its stability. Lower values indicate a higher likelihood of stability. Threshold values, typically up to ∼100 meV/atom, are used as stability cut-offs.^11^ In order to speed up electronic structure methods such as DFT, one possibility is to predict this quantity directly by using ML models, where compounds’ compositional and structural information is encoded and mapped directly onto Δ*H*_f_. This is otherwise known as composition prediction as it is used to identify which stoichiometries are stable by fixing structural variations. The ML models typically used include neural networks, kernel ridge regression, and random trees, while the training data are often taken from the OQMD, AFLOWlib, or Materials Project. For instance, models where the feature vector is only based on compositional information have been used to predict the stability of compounds forming a set of prototype structures (elpasolites, perovskites, heuslers, etc.), which is fixed for the compounds in the training set.^11−14^ Including structural information in the definition of a model mainly improves the predictions if large training datasets (>10^5^ data points) are used.^15^ Graph convolutional neural networks^16−18^ have notably been used to predict convex hull distances accurately and benefit greatly from structural features.^19,20^ Note that these can also be constructed with compositional information only.^21,22^ One downside to the inclusion of structural information in the models is that the optimized structure is not known prior to the search, so data for unrelaxed structures has to be used. This can notably be corrected by using ML interatomic potentials (MLIAPs), which are capable of performing relaxations.

MLIAPs combine atomic fingerprints, representing individual atomic environments in the form of feature vectors, with ML algorithms and effectively map the potential energy surface (PES) of a collection of atoms.^23^ The past decade has seen an immense expansion of the development and application of such potentials.^24−29^ When trained using active learning, MLIAPs have most notably been able to extend the length and time scales of *ab initio* molecular dynamic simulations by several orders of magnitude.^30−34^ Such potentials have been successfully applied to predict the energy and forces of alloys^35^ and have been used to accelerate and assist the construction or further exploration of binary and ternary convex hulls. Workflows built on these potentials use MLIAPs as surrogate models to first relax and then make energy predictions on a large library of prototype structures. The lowest energy structures are then compared to reference convex hulls obtained from DFT calculations. This process allows one to improve the reference convex hull diagram by identifying structures lying below it. The training of such potentials is crucial for adequate performance, and studies insist on using high-energy structures for relaxations to be reliable.

Work in this area has broadly been split into two categories. In the first, specific MLIAPs are trained for a given system,^36−42^ typically using active learning. In the second, MLIAPs are trained on large generic databases and are used to scan over many phases.^43,44^ The former is more accurate than the latter, but it is not transferable to other phases. Due to their higher accuracy, phase-specific MLIAPs can also be regarded as global structure optimizers, in that not only can they be used to identify specific stable compositions but they can also accurately predict their structure. Many other ML global structure optimizers exist, either in the form of novel workflows^45−48^ or by inserting MLIAPs into the pre-existing state-of-the-art global structure optimizers.^49−51^

In this work, we demonstrate how a MLIAP can be trained on data *readily available* on a mainstream repository, such as AFLOWlib,^6^ and used to screen a library of ternary-alloy prototypes constructed from their associated binary systems. Recently, we have shown^52^ that an ensemble of spectral neighbor-analysis potential (SNAP)^26^ models, trained on the energy data of the three binary subsystems associated with a ternary one, was able to predict the energies of ternary compounds with a mean absolute error (MAE) of ∼30 meV/atom, as long as the structures were fully relaxed. This not only provides a fast energy-screening tool for ternary compounds, which only requires existing *ab initio* data on binary structures, but also gives valuable insight into the fact that chemical environments within binary and ternary transition-metal alloys are similar. Such observation is at the heart of the workflow introduced here. A selection of binary structures, those close to their respective convex hull tie-planes, are selected as templates for ternary alloys. In a high-throughput setup, these are screened using an ensemble of SNAP models trained on binaries. The lowest-energy compounds are then selected as the most promising candidates, and their energies are calculated using high-fidelity DFT. The ternary convex hull is thus updated.

What makes this workflow different to tailor-made MLIAPs used for convex hull construction is that all the data, both for prototype generation and for training the SNAPs, is taken from the relevant binary phases of the AFLOWlib database.^6^ In other words, there is no need to generate any new data for the purpose of training the MLIAPs. Despite its training database not being specifically made, either by including important configurations through physical intuition or through active learning, it still has a low enough error on energy predictions to enable a high-throughput search of novel alloys. This is because stable binary and ternary phases, at least for the materials class of transition-metal intermetallics investigated here, share similar local atomic environments. In some sense, the workflow enables an interpolation of the data already available in AFLOWlib to scan ternary convex hulls and identify stable compositions. Since only a few high-energy structures and no out-of-equilibrium configurations are included in the SNAP training dataset, additional features are introduced in the workflow to increase the robustness of the predictions. These include constraints on the SNAP-driven relaxation (constant volume and the inclusion of a maximum number of steps) as well as using an ensemble of models.

In this paper, the workflow used to generate novel ternary compounds is presented. The methodology Section 2 details how ternary prototype structures are built from their binary counterparts and how binary compound data from AFLOWlib^6^ is used to train an ensemble of SNAP^26^ models. Such SNAPs are used to relax and screen ternary prototypes. Then, the results Section 3 present how the workflow is used to find stable phases for the Cu–Ag–Au and Mo–Ta–W ternary systems. The so-constructed convex hull diagrams are subsequently compared with their available AFLOWlib counterparts, and conclusions are drawn in Section 4. Finally, Section 5 presents the computational methods employed.

## 2 Methods

**Figure 1:**
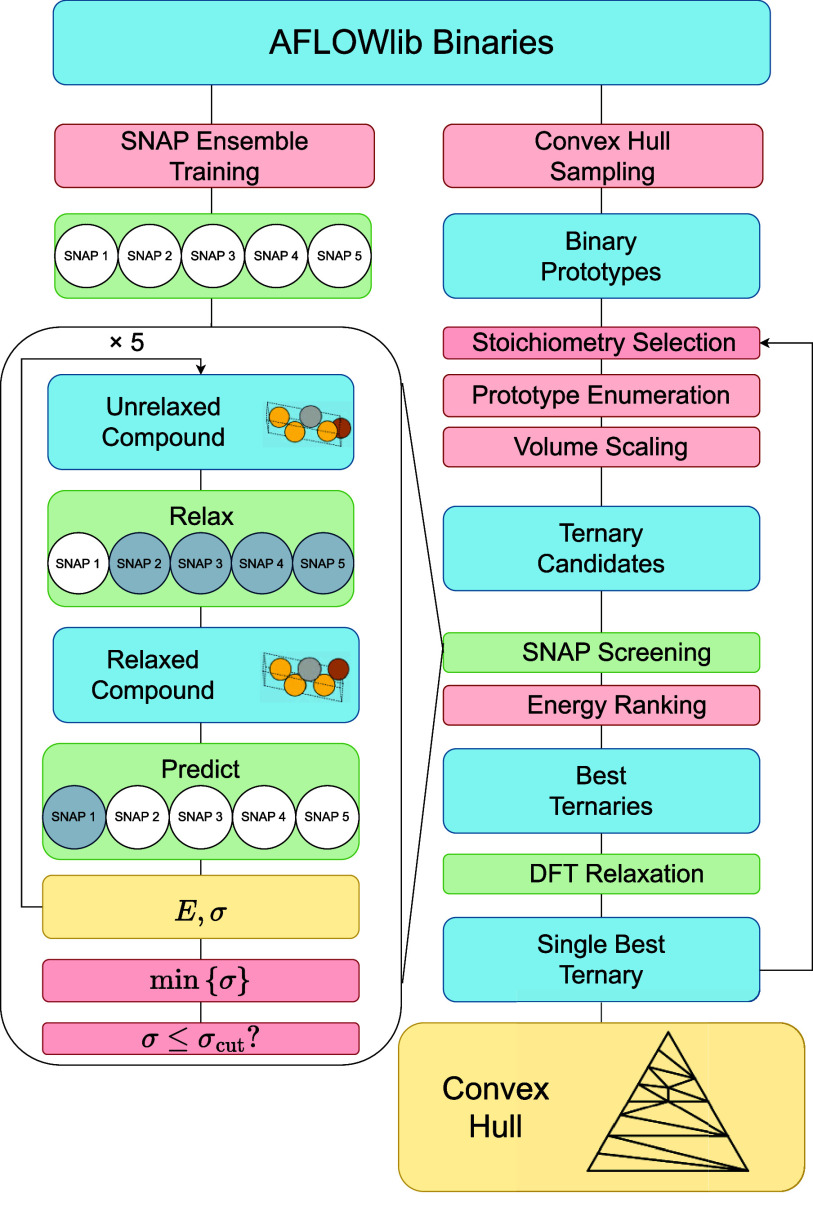
Diagram of the full stable ternary compound search workflow implemented in this work. Data available from the three binary subsystems associated with a ternary one (box at the top) is used for two tasks: (i) the training of an ensemble of SNAP models and (ii) the construction of a library of parent prototype structures. Derivative structures of the latter are created and all possible ternary decorations of these are produced. Each of them is then relaxed with the SNAPs model and the lowest-enthalpy structures are screened.

The general methodology of our workflow, schematically introduced in Figure 1, is described in detail. From the AFLOWlib database of binary compounds and their associated DFT-computed energies, an ensemble of SNAP models is trained. A subsection of these structures, the ones with the lowest enthalpy of formation, are also used as parent structures and form a library of prototypes. Note that here, as is standard practice in many DFT-based convex hull constructions, a compound’s enthalpy of formation is approximated solely by its ground-state DFT energy. As such, the terms enthalpy and energy will be considered equivalent throughout the rest of the study. Candidate ternaries are then created by generating all possible and unique derivative structures of such prototypes^53^ at a fixed composition,^54^ up to a maximum total number of atoms. These two parts are then combined as the ensemble of SNAP models is used to relax the candidate ternaries. The final energies are predicted through cross-validation (CV) within the ensemble of models, while the standard deviation of the predictions is also used to detect and remove geometries for which the relaxation has failed. The resulting structures with the lowest energy and standard deviation are selected as the best candidates (closest to the convex hull). Finally, full *ab initio* relaxation is performed for these. The Cu–Ag–Au ternary system is used to develop the methodology and is employed here as an example in each subsection.

### 2.1 Generating Prototypes

**Figure 2:**
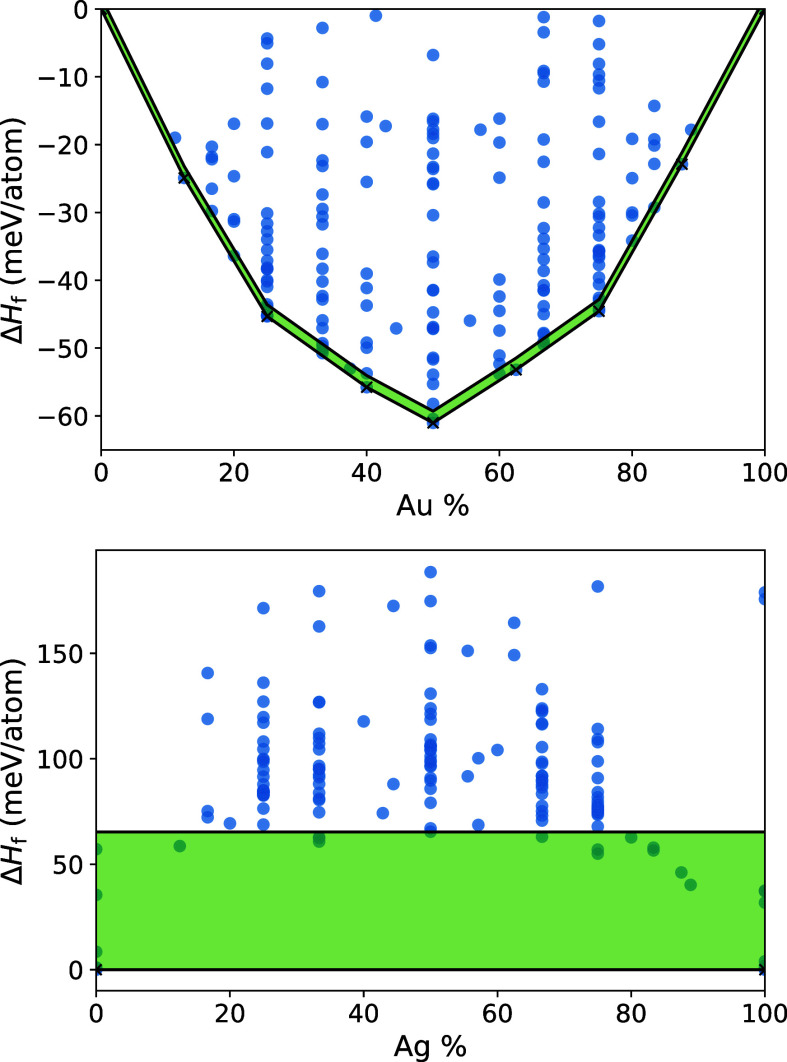
Convex hull diagrams of the Ag–Au (top) and Cu–Ag (bottom) binary systems. Each convex hull is defined by the lower black tie-lines, while the green-shadowed regions up to the higher full lines show the energy windows chosen to select the binary structures.

The first step of the workflow consists of creating a suitable library of ternary prototype materials. The driving idea of this work is that the local atomic environments seen in binary intermetallic alloys are similar to those in the associated ternaries, especially for structures close to equilibrium.^52^ In the context of the current work, this insight leads to choosing the binary structures as prototypes for the ternaries. More specifically, the bottom of the three binary convex hulls associated with a ternary system (in our example, Ag–Au, Cu–Ag, and Cu–Au for Cu–Ag–Au) are scanned to select the lowest-enthalpy compounds. Those within a certain energy range from the convex hull are then selected. All binary structures considered here are taken from the AFLOWlib database.^6^ The threshold energy selected differs depending on the system at hand to ensure that roughly the same number of structures are taken from each binary diagram. For instance, in our test system, Cu and Ag are immiscible.^55^ Therefore, all binaries have a positive enthalpy of formation and lie far from the Cu–Ag tie-line between the two elementary phases (*fcc* Cu and Ag). As a consequence, the energy window above the hull for this binary is larger than that of the other two. The convex hulls of Ag–Au and Cu–Ag are compared in Figures 2, and Table 1 gives the energy window used, as well as the number of structures selected for each binary system.

**Table 1:** Number of Structures, *N*_struct_, Selected from Each Binary System, *X*–*Y*, to Construct the Ternary Prototypes^a^

*X*–*Y*	*N*_struct_	Δ*E* (meV)
Ag–Au	24	1.7
Cu–Ag	25	65.4
Cu–Au	25	6.2

a Here, we also report the energy window, Δ*E*, above the convex hull used for the selection.

Once the prototypes are selected, the constituent atoms are stripped of their chemical identity, and all structures are compared using the AFLOWlib symmetry tool^56^ in order to curate redundancies. This is necessary since certain structure types (such as *fcc* or *bcc*) may be present several times in the collected database but may be “decorated” in several different ways for different stoichiometries and binaries. At this stage, all structures are reduced to their primitive cells. It is also important to note that single-element structures are also included in this analysis. This leads to a library of unique, undecorated prototypes, taken from the binary convex hulls. For the Cu–Ag–Au system, this results in 40 prototypes. Information on the prototype structures is provided in the Supporting Information.

From this set of prototypes, ternary alloys are generated. This task is performed at a fixed stoichiometry and for cells up to a maximum number of atoms, *N*_max_. For all the prototypes with a number of atoms compatible with the fixed stoichiometry, the set of all unique derivative structures are created by following the procedure introduced in refs (53), (54), (57), and by using the associated open source ENUMLIB code. The initial implementation of the algorithm begins from a parent lattice and uses group theory to efficiently enumerate all the unique ways to occupy the sites of supercells constructed from that lattice.^53^ Further modifications of the scheme allow for the starting structures to be defined by a lattice, an atomic basis (multilattices),^57^ and for the generation of derivative geometries at a fixed stoichiometry.^54^ This completes the first step of the workflow (blocks in the top right corner of Figure 1) and leads to a set of unique ternary compounds inspired by the structures of the binaries. The energy of these is then screened using a MLIAP.

### 2.2 Ensemble of SNAP Models

MLIAPs typically assume that the total energy, *E*, of a *N*-atom system defined by coordinates **r**^*N*^ can be written as a sum of atomic energies *E*_*i*_
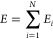
1

Such a partition, first proposed by Behler and Parrinello,^25^ is based on the principle of near-sightedness.^58,59^ The MLIAP of choice for this work is SNAP,^26^ which has proved to perform well regardless of the nature of the chemical bond.^60^ In this model, the total energy of a compound is written as a sum of linear combinations of the feature vectors describing the chemical environments of each atom *i* of the type α_*i*_ in the system. SNAP then takes the bispectrum components, , as feature vectors. The function, *E*_SNAP_, that returns the SNAP-predicted total energy is thus defined as
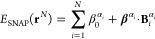
2where  and **β**^α^_*i*_ are the species-dependent linear coefficients of the ML model. Further details on this potential can be found in Section 5. SNAP’s linear form allows one to obtain good performance with a small number of features, 56 per species in our case, and when trained on small datasets (≤10^3^ data points).^61^ Furthermore, the SNAP hyperparameters are easy to optimize since the range of optimal values for *J*_max_ (the maximum angular momentum of the bispectrum) and *r*_cut_ (the cutoff radius) is wide and consistent for accurate performance.^26,35,62^ In our experience, the optimization of the atomic weights, although generally useful, only leads to modest improvements.^52^

As for our previous study, an ensemble of SNAP models is used to increase the robustness of the predictions. Furthermore, this provides a means of estimating the prediction uncertainty.^52^ The ensemble is defined as a set of *K* functions, , where each SNAP model, defined by *E*_SNAP_^*k*^, is trained differently and hence has different linear coefficients. The predicted energy of a new system with atomic positions, **r**^*N*^, is defined as the mean prediction of the models, *E*, and its uncertainty is estimated from the standard deviation, σ, of *E*, namely
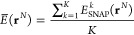
3
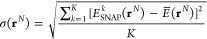
4

The training data only consists of binary alloys obtained from the AFLOWlib database. In the case of the Cu–Ag–Au system, the total energies have been recomputed for consistency by single-point DFT calculation (no further relaxation is performed). Differently from what was done when generating the prototypes, here all binaries, no matter their distance from the convex hull, are included in the training dataset. The same workflow is also used for Mo–Ta–W (the results are described in Section 3), for which we demonstrate that the energy values taken directly from AFLOWlib are suitable for training the SNAP models. The full details on the Cu–Ag–Au binary subsystems can be found in ref (52).

**Figure 3:**
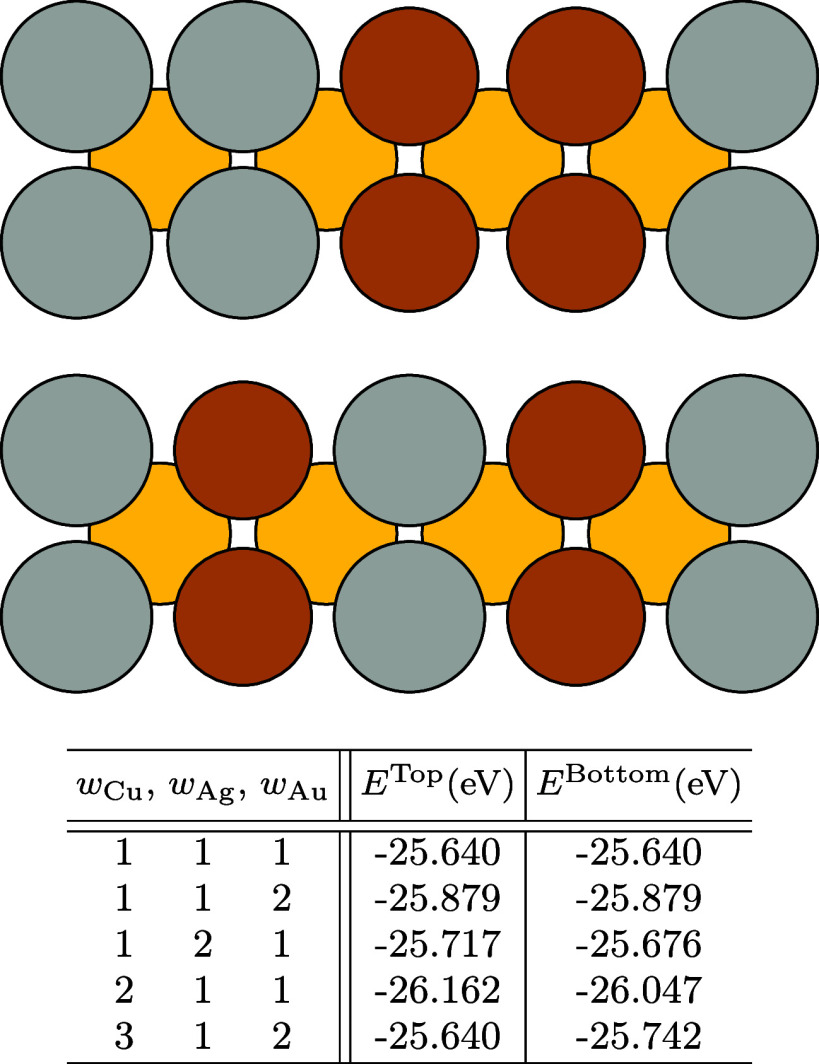
SNAP performance was assessed for two structurally identical prototypes. The upper two panels show two different possible site occupations for a 3 × 1 × 1 *bcc* derivative structure with Cu_1_Ag_1_Au_2_ stoichiometry. Here, we show the *z* axis view, with bronze, silver, and gold spheres representing Cu, Ag, and Au, respectively. The table shows the SNAP-predicted energies for the two prototypes when the SNAP is trained with different atomic weights, {*w*_α_}, as indicated in the first column. Note that when the Cu and Ag weights are identical, the two energies coincide by construction. All the crystal structure visualizations are generated with the use of the atomic simulation environment (ASE).^63^

Previously, 10 different SNAP models within the ensemble were obtained by training on different subsets of the same size of the binary-alloy database.^52^ For this work, 5 models are trained on the full database, but for each one, a different set of atomic weights for Cu, Ag, and Au are used to compute the bispectrum components. This difference is motivated by the need to distinguish compounds with identical site positions in their structure (e.g., the sites of a *bcc* supercell) but different atomic site occupations. If the atomic weights for all species are identical, for some of these structures, notably the high-symmetry ones, SNAP will predict identical energies for different compounds. This is illustrated in Figure 3 for two distinct compounds obtained as *bcc* derivative structures with the composition Cu_1_Ag_1_Au_2_. Prototypes A and B only differ by a permutation of the Ag and Cu atoms. Hence, SNAP models using identical weights for these two atomic types will fail to predict different energies for the compounds. Therefore, by construction, in the ensemble created, the two elements in each pair of atomic types (e.g., Cu and Ag in Cu–Ag) have different weights in at least one model.

Before selecting the values for the atomic weights, *r*_cut_ and *J*_max_ are optimized manually and independently by using 10-fold Monte Carlo CV for fixed identical weights, {1, 1, 1} and thus find *r*_cut_ = 3.5 Å and *J*_max_ = 4. For these values, the optimal atomic weights are set by performing a grid search, with the same CV method, where all three atomic weights are varied from −5 to 5 in steps of 1 (omitting 0). Within this search space, the sets of weights used for the SNAP models in the ensemble are chosen to minimize the CV root-mean squared error (RMSE). The training and CV errors for each model of the ensemble are given in Table 2. The ensemble is then used to predict which of the prototype structures has the lowest enthalpy.

**Table 2:** Training and CV Errors for the 5 Models, Defined by Different Atomic Weights {*w*_α_}, of the Ensemble^a^

*w*_Cu_	*w*_Ag_	*w*_Au_	training MAE	training RMSE	CV MAE	CV RMSE
1	1	2	8.0	13.4	27.1	83.5
1	2	2	8.7	13.5	24.8	64.7
–1	–2	–1	9.7	16.4	30.6	86.4
–1	–2	–2	8.5	13.2	23.5	64.3
–1	–1	–2	7.7	13.1	25.6	75.0

a Here, we report the MAE and the RMSE. All values are given in meV/atom.

### 2.3 Energy Screening

The final aim of the workflow is to suggest low-energy ternary structures with a given stoichiometry. Since many compounds with a large energy spread are screened, the suggestions made need to be accurate (must include low-energy structures) and reliable (must not include high-energy and unphysical systems). While the energy error of the SNAP surrogate model is low, it is still prone to making poor predictions about new systems that do not resemble the structures seen in training. As a result, the construction of the workflow focuses on the robustness of the final predictions made. Note that choosing parent prototypes from binary compounds increases the reliability of the predictions.

**Figure 4:**
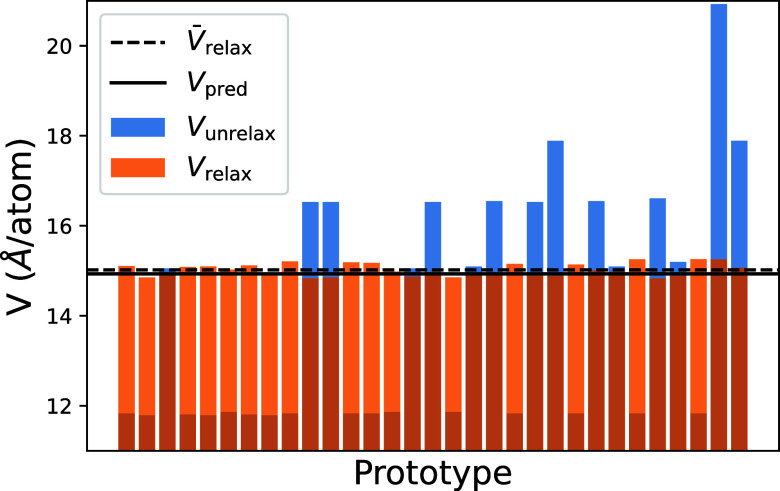
Plot showing the initial unrelaxed volumes, *V*_unrelax_, and relaxed equilibrium ones, *V*_relax_, of a set of ternary prototypes at the stoichiometry Cu_2_Ag_1_Au_1_. Unrelaxed volumes are chosen from the volume of the binary associated with each ternary compound’s structure. The dashed line indicates the mean equilibrium volumes for these compounds, , while the full line shows the volume predicted by the weighted average of the elemental volumes, *V*_pred_.

The first step in the energy-screening process consists of setting the compounds’ unit-cell volume. This is chosen by taking the weighted average of the elemental volumes of the constituent atoms, an approximation that reproduces the results from *ab initio*-relaxed compounds quite well, as is illustrated in Figure 4. Then, the volume and all lattice parameters are kept fixed during any relaxation driven by the SNAP models. This is because, while the training database includes a diverse set of structures, they are all at equilibrium, namely, their forces and stress-tensor elements are close to zero. Therefore, no configurations are strongly compressed or expanded, a fact that causes the SNAP models to perform poorly on the prediction of equilibrium volumes and lattice parameters. The volume is allowed to change only for the final, most promising structures selected for the DFT relaxation.

Each of the *K* SNAP models available is used to drive an ionic relaxation, with a maximum of *N*_s_ steps for all of the prototypes, leading to *K* differently relaxed structures per prototype. A “cross-validated” energy prediction is given for each relaxed structure. Given a candidate obtained by relaxing a prototype with the *k*-th SNAP model, the energy prediction is made with *K* – 1 models, namely, all SNAPs bar the one used for the relaxation of the candidate at hand. The mean and standard deviation of the energy predictions for the *K* – 1 models are then saved. For every prototype, one of the *K* relaxed structures obtained is selected, namely, the one with the lowest “cross-validated” standard deviation. This is the structure whose final total energy has received the largest consensus among the SNAP models. Therefore, there is only one relaxed structure per prototype. This procedure is illustrated in the flowchart in Figure 1.

The reason why this process is not a single ionic relaxation stems from the drive toward robustness of the predictions. Without the inclusion of the *N*_s_ iteration cutoff, some of the relaxations would lead to structures that are trapped in unphysical local minima of the PES of the driving SNAP model. By stopping the relaxation process at a low number of steps (*N*_s_ = 10 in this study), this effect is mostly avoided as the structures cannot change too drastically. For the relaxations that are accurately driven by SNAP, the largest drop in energy typically occurs during the first few steps of the relaxation process. While accurate relaxations are also cut before convergence, as they are not distinguished from the inaccurate ones, the final structures are still lower in energy than the initial prototypes. This reduces the likelihood of obtaining high-energy structures and the total run time of the workflow remains modest.

Using “cross-validated” energy predictions of the relaxed structures helps to remove the bias of specific SNAP models. The SNAP driving the relaxation typically predicts the final structure to have a lower energy than the initial one since it moves the geometric configuration toward an (at least) local minimum of *its* particular PES. If the relaxation is inaccurate, the resulting structure will be, in fact, high in energy (as predicted by DFT). The relaxation-driving SNAP model is, therefore, not used for energy predictions. Instead, the other models of the ensemble are used, since they are less likely to present a bias toward that relaxed structure and therefore to predict it being low in energy. Note that since the compounds used as training data are the same for all of the *K* models, they could also lead to biases for the same structure. This is accounted for by using the “cross-validated” standard deviation, rather than the mean energy, to select the “best” structure out of the *K* relaxed ones. Indeed, even if several/all SNAP models are biased toward a particular structure and collectively predict it to have a low energy, the inaccurate predictions of each model will be different. This is because they are inaccurate, extrapolated predictions. It has indeed been shown that the standard deviation of SNAPs correlates with the error of the ensemble’s prediction.^52^ Hence, while the mean prediction of the ensemble may give a low energy value, the standard deviation will be large.

Finally, the “cross-validated” standard deviation prediction for all structures must be lower than a cutoff value, σ_cut_, to be considered for the final energy screening. This typically excludes structures with low SNAP-predicted energy that DFT returns to be high-energy as well as structures with high SNAP-predicted energy. From the sample of structures selected, the ones with the lowest “cross-validated” mean energy are chosen and relaxed with DFT. In this study, 15 structures per stoichiometry are selected through such a process.

In summary, the workflow described creates a set of prototypes and uses an ensemble of ML potentials to relax and screen the structures, which are most likely to have low energy. This is done iteratively at fixed stoichiometries. The final selected compounds are then recomputed with full DFT relaxation. The workflow, therefore, allows one to perform all of the computationally intensive DFT calculations only on the most promising candidates. In the following sections, this workflow will be used to reconstruct the ternary-alloy convex hulls of Cu–Ag–Au and Mo–Ta–W.

## 3 Results

This section, which is separated into two subsections, presents the key outcomes of our method. First, we examine the performance of our workflow against the well-established and extensively studied Cu–Ag–Au phase diagram.^64^ Then, we provide a comparison between our results and those of one of the better-characterized phase diagrams available in AFLOWlib, specifically Mo–Ta–W. By benchmarking the workflow phase diagram predictions to the DFT created ones available in AFLOWlib, we gain valuable insights into the effectiveness of our approach.

In order to accurately evaluate the stability of our predicted prototypes and ensure consistency in our analysis, we have used the QHull(65) library to calculate the convex hulls presented in this work. The stable, ground-state compounds used to construct the reference convex hull (a subset of the full database) were downloaded from AFLOWlib. For both ternary systems studied, in order to guarantee consistency, we have recalculated the energies of these compounds with the Vienna Ab initio Simulation Package (VASP).^66^ Throughout the entire process, we have strictly followed the AFLOWlib standards as outlined in ref (67), with an energy cutoff of 600 eV to ensure tight convergence. More information regarding the DFT calculations can be found in Section 5.1. It should be noted that, for the Mo–Ta–W ternary system, we directly use the AFLOWlib precomputed energies for the training of the SNAPs ensemble. For the Cu–Ag–Au system, energy data are taken from a previous project,^52^ where they were recalculated with VASP.

### 3.1 Cu–Ag–Au Ternary Convex Hull

In order to evaluate the performance of our workflow, it is essential to select a well-studied phase diagram that meets specific requirements. Another key consideration is the availability of sufficient data to train an accurate MLIAP. To facilitate the identification and correction of any errors during the development of the workflow, it is also beneficial to choose a relatively simple phase diagram. With these criteria in mind, we chose the Cu–Ag–Au ternary system, a choice further supported by the fact that the MLIAPs for this phase diagram have already been optimized and trained in our previous work.^52^

**Figure 5:**
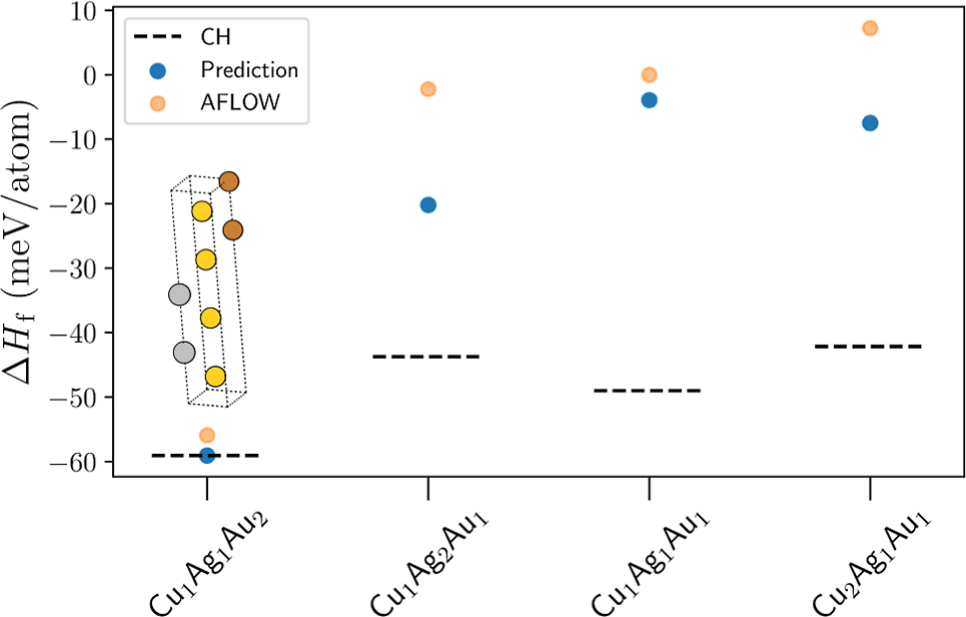
Workflow predictions for the Cu–Ag–Au ternary system across different stoichiometries. Graph presents the different compositions and their corresponding enthalpies of formation, Δ*H*_f_. The blue points are associated with the predictions from the proposed workflow, whereas the orange ones represent the lowest-energy AFLOWlib points. Dashed line (CH) marks the tie-plane position of the convex hull. Proposed workflow manages to identify one stable intermetallic phase among these, namely, Cu_1_Ag_1_Au_2_. Furthermore, it manages to outperform the AFLOW dictionary method in all of the presented cases. Unit cell of the newly discovered crystal structure on the convex hull is presented as well. Here, Au atoms are in gold, Ag in silver, and Cu in bronze.

As a proof of concept, we have focused on the equiatomic Cu_1_Ag_1_Au_1_ ternary phase as well as phases with stoichiometric ratios of 2–1–1^a^ and 2–2–1. The reason for this choice is that data at these stoichiometries is available in AFLOWlib for comparison. The results of the workflow are presented in Figure 5 and Table 3. In order to quantitatively assess the stability of the structures proposed by the workflow, we use δ, the distance from the reference convex hull (AFLOWlib). A negative value indicates that the predicted structure lies below the calculated convex hull, establishing its stability as an intermetallic compound. Then, the convex hull needs to be recalculated and corrected by taking into account the newly predicted stable structure. In contrast, a positive distance from the convex hull provides a criterion for assessing whether the structure is metastable or unstable. In Table 3, values predicted by the workflow (AFLOWlib) are labeled as δ^WP^ (δ^AFLOW^).

**Table 3:** Workflow Predictions for the Cu–Ag–Au Ternary System with 2–2–1 and 3–1–1 Compositions^a^

stoichiometry	δ^AFLOW^ (meV/atom)	δ^WP^ (meV/atom)
Cu_2_Ag_2_Au_1_	208.95	25.99
Cu_2_Ag_1_Au_2_	205.69	37.45
Cu_1_Ag_2_Au_2_	90.27	17.21
Cu_3_Ag_1_Au_1_	–	20.35
Cu_1_Ag_3_Au_1_	–	31.05
Cu_1_Ag_1_Au_3_	–	–0.02

a The stoichiometries and their corresponding distances from the convex hull, δ^WP^, are presented. For the 2–2–1 compounds, the distances from the convex hull of the phases available in the AFLOWlib database, δ^AFLOW^, are also given. Note that for all materials, the distance from the AFLOWlib convex hull tie-plane is used as a reference. A new gold-heavy intermetallic, namely, Cu_1_Ag_1_Au_3_ is predicted to be stable (see Figure 6).

The scalability and speed of the algorithm allow us, in principle, to investigate more regions of the phase diagram in a single study than a pure DFT phase diagram construction scheme. This is exemplified by using the proposed workflow to predict structures that are not in AFLOWlib’s database, namely, compounds with 3–1–1 stoichiometry. The results of the benchmark are presented in Table 3, alongside the crystal structure of the new stable phase, Cu_1_Ag_1_Au_3_, in Figure 6.

**Figure 6:**
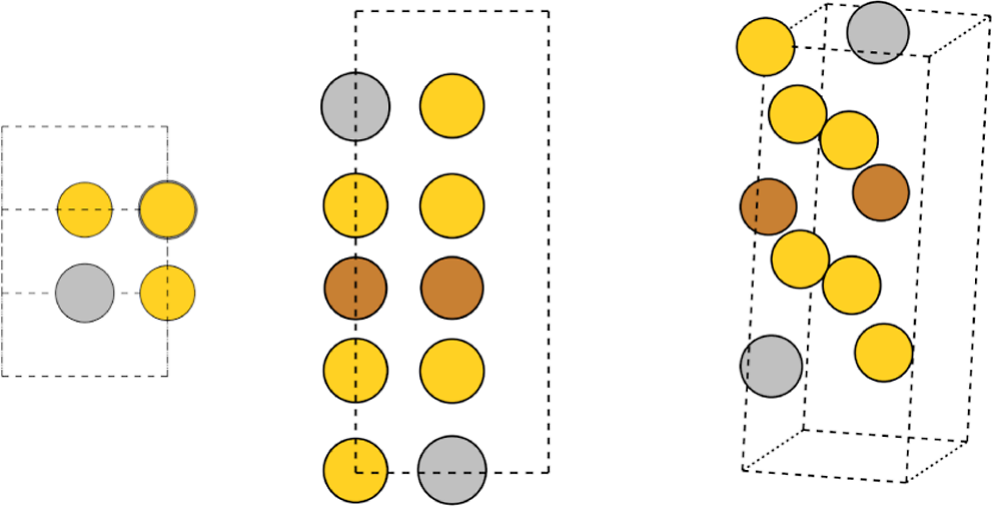
Unit cell of the crystal structure found on the convex hull at the composition Cu_1_Ag_1_Au_3_ is presented in both a top view with respect to the *z* axis (left), a side view along the *x* axis (middle), and a tilted view (right). In this structure, Au atoms are colored in gold, Ag atoms in silver, and Cu atoms in bronze.

Our approach outperforms the AFLOW dictionary method in all cases, demonstrating a better predictive capability, which arises from the exploration of a larger pool of prototypes. Interestingly, the structures predicted by the proposed workflow are consistently closer to the convex hull than those predicted by the AFLOW dictionary method. This is to be expected since the workflow effectively selects the relevant structures for creating the pool of ternary candidates. Furthermore, our model consistently predicts structures with a negative or almost negative (<10 meV/atom) enthalpy of formation, a fact that gives us confidence in the reliability of the predicted structures. Notably, we have been able to identify two new gold-heavy stable phases, namely, Cu_1_Ag_1_Au_2_ and Cu_1_Ag_1_Au_3_. This indicates that stable intermetallic phases may exist on the gold side of the phase diagram. We have confidence in our prediction, given the fact that the dictionary method structure for Cu_1_Ag_1_Au_2_ is within 3 meV/atom of the convex hull, suggesting the possibility of the existence of a stable phase. This is consistent with the formation of the solid solutions in the gold-rich region of the experimental phase diagram.^64^ The rest of the structures are considered to be potentially metastable, with an average distance from the convex hull of around 30 meV/atom.^68^ Overall, our analysis demonstrates the ability of the workflow introduced here to predict structures closer to the convex hull than those from the state-of-the-art dictionary method and possibly uncover novel phases should they exist.

### 3.2 Mo–Ta–W Ternary Convex Hull

As a second benchmark, we explore a phase diagram that exhibits a variety of stable phases. Thus, the main criterion for our selection, among all of the possible transition-metal ternary combinations, is the total number of stable compounds. The Mo–Ta–W ternary system emerged as a good candidate based on a search run with the AFLOW REST-API.^69^ In fact, it exhibits the highest number of stable ternary phases of the entire database of transition metal alloys. In order to compare our proposed workflow with the dictionary method, we have made predictions corresponding to the same stoichiometries presented in the previous section. Furthermore, we used our method to explore areas of the phase diagram poorly covered in AFLOWlib.

We now perform a similar analysis to that described in the previous section. The structure prototypes used for element decoration are extracted from those of the binaries closest to their respective convex hulls. Information on the prototype structures is provided in Table S2 in the Supporting Information. Then, an ensemble of ML models relaxes the created structures and orders them based on their predicted energy. A set of 15 structures for each stoichiometry, corresponding to those with the lowest predicted energies, is sampled and proceeded to the next stage. The latter consists of performing a DFT relaxation and a static calculation for each one of these predictions. A significant difference with respect to the Cu–Ag–Au system is that, we now use AFLOWlib’s database to train the models without further recalculation. The AFLOW REST-API is used to download the energies and the crystal structures for the three binary convex hulls (Mo–W, Ta–W, and Mo–Ta). The models are trained as explained in the Methods Section (see Section 2). Recycling data already available on AFLOWlib allows us to avoid about 1500 DFT relaxation calculations, some of them for cells up to 46 atoms, just for the training of the model.

**Figure 7:**
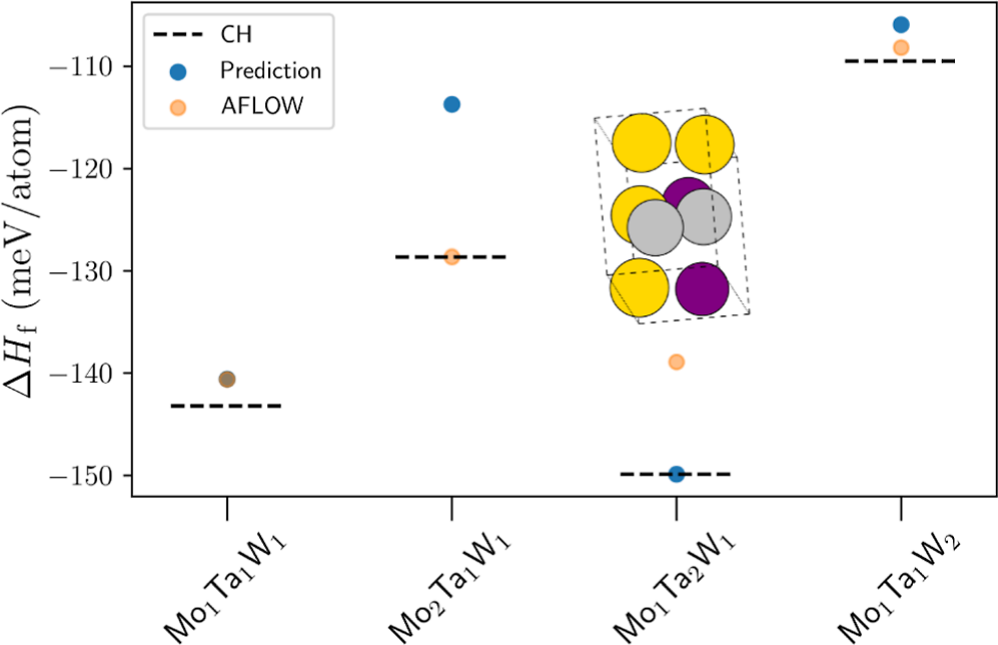
Workflow predictions for the Mo–Ta–W ternary system across different stoichiometries, 1–1–1 and 2–1–1. Graph presents the different compositions and their corresponding enthalpy of formation, Δ*H*_f_. The blue points are associated with the predictions from the proposed workflow, whereas the orange ones represent the lowest-energy AFLOWlib data. Dashed line marks the tie-plane position of the convex hull (CH). The proposed workflow has managed to identify one previously unknown intermetallic phase, namely, Mo_1_Ta_2_W_1_, whose unit cell is shown as an inset. Here, Mo atoms are in purple, Ta in gold, and W in silver.

The results for the 1–1–1 and 2–1–1 compositions, those with stable phases in AFLOWlib, are presented first in Figure 7. In this case as well, we predict a new stable intermetallic phase, Mo_1_Ta_2_W_1_. However, this time, our workflow does not consistently outperform the dictionary method. In fact, for two out of the four stoichiometries investigated in Figure 7, we obtain compounds with energies similar to the ones already present in AFLOWlib, while for one, Mo_2_Ta_1_W_1_, our search delivers a compound with a higher energy. Interestingly, in this last case, our newly found structure and the original one, contained in AFLOWlib, belong to different space groups. The AFLOW-predicted one has space group 107 (tetragonal), while our scheme finds a low-symmetry monoclinic crystal structure with space group 9. The final geometries are not equivalent as determined by the AFLOW-SYM tool.^56^ Nevertheless, the compound discovered with the workflow only has an enthalpy of formation 14.91 meV/atom higher than that of the AFLOWlib compound.

As a force field-based approach, our workflow gets better when the MLIAP improves. In this case, we have extracted the data used to train the SNAPs from the AFLOWlib repository, a detail that led to a force field less accurate than that used for the Cu–Ag–Au system. In fact, minor inconsistencies in the energy data, due to unconverged results, may generate errors in the force field.^70,71^ That being understood, we have still demonstrated that new phases can be predicted by an almost DFT-free workflow since our initial data for model training are readily available in the AFLOWlib database. The workflow systematically assesses a wide range of compositions and potential compounds. Specifically, it involves the evaluation of 331,734 ternaries based on their calculated SNAP energies. Following this, the 15 lowest-enthalpy structures, for each stoichiometry, undergo relaxation with DFT. Interestingly, the DFT analysis reveals that, on average, the most stable compound ranks seventh among the suggested options. Additionally, the *ab initio* computations are shortened since all compounds move closer to their equilibrium geometry after the SNAP-guided relaxation, in contrast to their fully unrelaxed counterparts.

Perhaps a more accurate force field would also be able to find the AFLOWlib minimum for Mo_2_Ta_1_W_1_ (see Figure 7). Nevertheless, our workflow is already able to identify the majority of the structures close to the convex hull. It should also be noted that this is the phase diagram for which AFLOWlib’s dictionary method works best, as it is able to detect four intermetallic phases, more than for any other transition metal alloy phase diagram.

Then, we move on to analyze stoichiometries poorly explored in AFLOWlib, namely, 2–2–1 and 4–1–1. In Table 4, we provide a comparison of the distance from the convex hull for the structures predicted with our method, δ^WP^, and the ones from AFLOWlib, δ^AFLOW^. For these compositions, the AFLOWlib compounds are unstable, as they all have a positive enthalpy of formation. In contrast, those found by our workflow all have a negative enthalpy of formation and are found near or at the convex hull. These results provide a comparison between our method and that of AFLOWlib for structures predicted to be unstable by the latter.

**Table 4:** Workflow Predictions for the Mo–Ta–W Ternary System with 2–2–1 and 4–1–1 Compositions^a^

stoichiometry	δ^AFLOW^ (meV/atom)	δ^WP^ (meV/atom)
Mo_2_Ta_2_W_1_	880.90	0.00
Mo_1_Ta_2_W_2_	962.84	0.00
Mo_2_Ta_1_W_2_	1032.50	8.50
Mo_4_Ta_1_W_1_	320.95	46.56
Mo_1_Ta_4_W_1_	516.30	3.25
Mo_1_Ta_1_W_4_	334.16	0.00

a The stoichiometries and their corresponding distance from the convex hull, δ, are presented (δ^WP^ is for compounds generated by our workflow, while δ^AFLOW^ is for the AFLOWlib compounds). Three intermetallic phases are predicted to be stable and two others are metastable. Surprisingly, our algorithm is able to find structures with an energy of up to 1 eV/atom lower than that identified by the dictionary method of AFLOWlib.

The ability of our workflow to consistently predict structures that (i) are close to the convex hull and (ii) have a negative enthalpy of formation is thus demonstrated. The former point means that we have an effective algorithm to use for the structure search in regions of interest. The latter validates our physical intuition behind the assumption that the crystal structures of the binary alloys close to the convex hull can be used as a template for atomic decoration in the search for ternary phases. This approach has allowed us to identify three new intermetallic compounds (see Table 4), namely, Mo_2_Ta_2_W_1_, Mo_1_Ta_2_W_2_, and Mo_1_Ta_1_W_4_. Such positive results demonstrate the value of the enhanced freedom in the structure search provided by our algorithm with respect to the dictionary methods.

**Figure 8:**
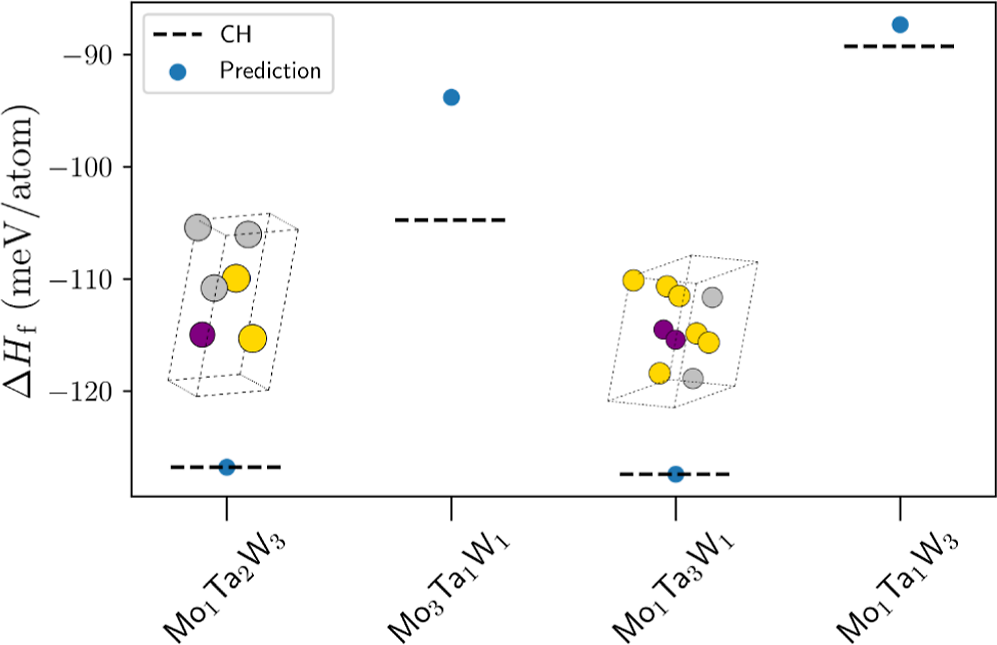
Workflow predictions (blue points) of the enthalpy of formation for the Mo–Ta–W ternary system across the Mo_1_Ta_2_W_3_ and 3–1–1 compositions. Enthalpy of formation for each composition at the appropriate convex hull tie-plane (CH) is shown as a dashed line. Unit cells of the crystal structures found on the convex hull are presented as well. Here, Mo atoms are colored purple, Ta gold, and W silver. Two new intermetallics alloys have been identified, namely, Mo_1_Ta_2_W_3_ and Mo_1_Ta_3_W_1_.

Finally, following the same spirit as that for the analysis of the Cu–Ag–Au system, we now turn our attention to previously unexplored areas of the ternary convex hull. Our results for the Mo_1_Ta_2_W_3_ and 3–1–1 compositions are shown in Figure 8.

**Figure 9:**
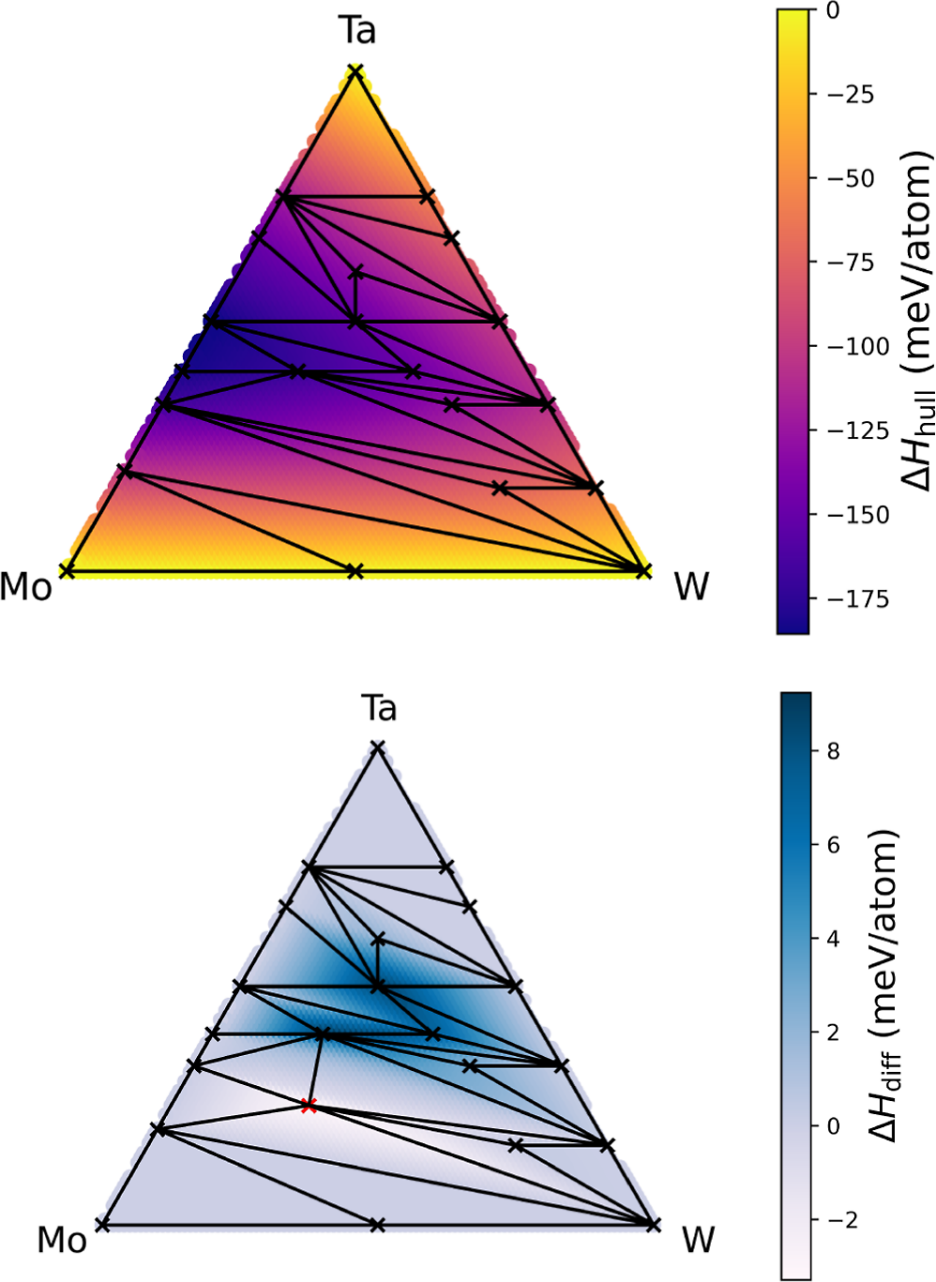
Workflow-computed convex hull for the Mo–Ta–W system (upper panel). Color heat map corresponds to the calculated enthalpy of formation at a given stoichiometry. In the lower panel, we present the difference between the convex hull of AFLOWlib (reference) and that computed by our workflow. Black crosses for the ternary region symbolize the newly predicted intermetallic phases, and the red cross denotes the only stable intermetallic that was originally predicted by the AFLOW dictionary method.

As one can observe, together with structures away from the tie-plane, we also find two new stable compounds, namely, Mo_1_Ta_2_W_3_ and Mo_1_Ta_3_W_1_. Such new phases, together with the low-energy ones previously discussed, call for a modification of the ternary convex hull that exists in AFLOWlib. The new diagram is presented in the top panel of Figure 9. In order to facilitate the comparison, the lower panel of the same figure shows the difference between the AFLOW- and our workflow-predicted convex hulls (positive values mean that our predicted convex hull is lower in energy than the original AFLOWlib one).

The new convex hull returns a picture where most of the stable ternary structures identified belong to the Ta–W heavy area and only one intermetallic alloy exists in the Mo-rich region of the compositional space. The latter is the compound found on AFLOWlib. Interestingly, the new phases predicted by our workflow undercut Mo_1_Ta_1_W_1_, Mo_1_Ta_2_W_1_, and Mo_1_Ta_1_W_2_, the other intermetallic alloys initially predicted as stable by AFLOWlib. These are now, respectively, 2.59, 10.95, and 1.31 meV/atom, above their associated tie-planes, and have to be considered metastable. Experimentally, there is evidence that the Mo–Ta–W system forms a ternary solid solution^72^ at finite temperature across the entire phase diagram. It should be noted that the Mo–Ta binary space is far better sampled by AFLOWlib than the Ta–W and Mo–W ones. This could imply that it is more difficult to reach the convex hull close to such a facet of the diagram. In contrast, the Mo–W system only displays small enthalpies of formation for the stable binary phases, implying that both Mo and W form more stable phases with Ta than among themselves. These two reasons could explain why it is more difficult to find stable intermetallic phases in the Mo-rich part of the composition space.

In summary, the workflow developed provides a comprehensive scan across the ternary composition space, enabling the construction of a convex hull with DFT-accuracy and identifying areas prone to alloy stability through the discovery of new hull points. This is accomplished by screening on the order of 10^5^ candidate compounds with the SNAPs ensemble. In the case of the Cu–Ag–Au test system, two stable Au-rich compounds are found, which are not present on the AFLOWlib convex hull. The new Cu_1_Ag_1_Au_2_ (Figure 5) and Cu_1_Ag_1_Au_3_ (Figure 6) ternary structures have space groups 123 and 63, respectively. They resemble distorted *bcc* and *hcp* structures. The workflow hence highlights the region in which Cu–Ag–Au alloys are likely to form. The experimental structures for these phases are *fcc* solid-state solutions.^55,64,73^ For the Mo–Ta–W system, six novel compounds are identified, undercutting three of the AFLOWlib compounds, leaving only the Mo_2_Ta_1_W_1_ intermetallic to lie on the convex hull. Experimentally, *bcc* solid-state solutions form across the full compositional range.^72^ One of the phases discovered, Mo_1_Ta_1_W_4_, possesses a tetragonally distorted *bcc* structure, while the others have space groups 74 (Mo_1_Ta_2_W_1_), 12 (Mo_1_Ta_2_W_2_ and Mo_1_Ta_3_W_1_), and 38 (Mo_1_Ta_2_Ta_3_ and Mo_2_Ta_2_W_1_). The results found here suggest that at low temperatures, the Mo-rich corner is dominated by an intermetallic phase, Mo_2_Ta_1_W_1_. However, the regions with lower Mo concentrations form many more different intermetallics that lie close to each other in energy. This region is thus more susceptible to the formation of solid-state phases at finite temperatures. The convex hulls obtained with this workflow suggest promising regions of material stability, notably in the form of solid-state solutions for the two systems studied. This could help guide experimental studies of the synthesis of stable ternary alloys. Our strategy resembles the MatLearn^74^ approach in spirit but is a more accurate method as it provides DFT-level convex hulls. This is illustrated by the fact that no novel ternary phases are predicted by MatLearn for Cu–Ag–Au and Mo–Ta–W, and for the former, the only “known” phase (from DFT) is in the Cu-rich region, which differs from the experimental phase diagram.

## 4 Conclusions

We developed a workflow that predicts the crystal structure and assesses the stability of ternary compounds of a particular stoichiometry. A library of prototype structures is formed from the lowest-enthalpy alloys of the associated binary subsystems. From this database, derivative ternary structures are generated by site decoration. Then, an ensemble of SNAP force fields is used to select the most promising structures among them, bypassing the majority of the *ab initio* calculations. Therefore, the proposed workflow highly increases the throughput in the search for stable ternary compounds without compromising the quality of the predictions. This is used here to map the ternary convex hull of the transition-metal alloys. The crucial aspect of the proposed scheme is that both the training of the force fields and the creation of the prototype ternary structures are based solely on knowledge of the binary phases. As such, no additional DFT calculations are required since both the structures and their corresponding energies are readily available on the AFLOWlib database. Employing *ab initio* calculations solely in the final stage of the workflow and focusing them on the most promising candidates allows us to perform a comprehensive exploration of the phase diagram of a ternary system with only a few hundred DFT calculations. This enables us to map previously unexplored portions of the ternary space and identify regions of interest, thus driving the discovery of novel compounds.

We have demonstrated that the proposed workflow is able to predict crystal structures with negative enthalpy of formation and effectively identify the stable intermetallics should they exist. In particular, we used the Cu–Ag–Au and Mo–Ta–W ternary systems as an example. In the first case, we have predicted several new phases that, although not all thermodynamically stable, have an enthalpy of formation lower than those found by the AFLOW dictionary method. In addition, we have identified a Au-rich composition region where stable intermetallic phases are expected, in accordance with the location of solid solutions in the experimental phase diagram.^64^ Interestingly, in the case of Mo–Ta–W, one of the ternary systems with the largest number of stable intermetallics in AFLOWlib, our method is capable of identifying a plethora of new phases, resulting in the correction of the original DFT-calculated convex hull proposed by AFLOW.

In summary, we have developed a novel way to integrate ML with a DFT workflow. Although the ML model introduced here does not perform as well as force fields with tailor-made databases, its construction requires no new DFT calculations and simply recycles pre-existing results already present in large-scale databases. This represents an example of how ML interatomic potentials can be seamlessly integrated into a materials design pipeline without the need to generate ad hoc large training sets.

## 5 Computational Methods

The details of the computational methods are presented in this section. The parameters used for the DFT calculations run with VASP^66^ are first discussed. A brief presentation of the SNAP^26^ is then given, along with details of the implementations used for the current work.

### 5.1 DFT Calculations

All DFT calculations are performed using the VASP,^66^ version 5.4.4. Projector augmented wave (PAW) pseudopotentials are used for each element together with the Perdew–Burke–Ernzerhof (PBE) functional.^75^ A plane wave cutoff of 600 eV is used for all calculations. The energy convergence criterion for each self-consistent cycle is of 10^–4^ eV. Full atomic relaxations are performed (update of atomic positions, cell volume, and lattice parameters) with a stopping criterion on the forces of 10^–3^ eV/Å. A Fermi–Dirac smearing of 0.2 eV is chosen for all calculations.

For the *k*-point sampling, a gamma-centered mesh is employed for all calculations. The density of the mesh and the spacing between *k*-points is chosen based on AFLOWlib’s convergence criteria.^67^ The mesh is system-specific and is determined from the *N*_KPPRA_ (number of *k*-points per reciprocal atom). The number of sampling points along each direction is proportional to the norm of the corresponding reciprocal lattice vector. The total number of sampling points per reciprocal atom is then minimized and *N*_KPPRA_ is used as a lower bound. Values of 10 × 10^3^ and 6 × 10^3^ are used for static calculations and relaxations, respectively.

### 5.2 Spectral Analysis Neighbor Potential

SNAP^26^ is used as an energy predictor. As described in Section II B, an ensemble of models is employed for predictions. Equation 2 defines the expression of the function, *E*_SNAP_, and combined with eq 1, gives the energy of a system with *N* atoms. The atomic fingerprints that define the chemical environments of each atom *i* in the system, belonging to species α_*i*_, are the bispectrum components.^23^ These are used to represent configurations instead of seemingly more obvious choices (e.g., atomic Cartesian coordinates), as they are invariant upon rotation and permutations of identical atoms. Note that invariance with respect to translations is guaranteed by eq 1. For each atom, the vector , which collects the first components up to a maximum index, is taken as a feature for the ML model (ridge regression in the case of SNAP). A short description of the bispectrum components is given below.

The neighborhood of an atom *i* atom can be described by a density function, ρ_*i*_, centered at that atom, with delta functions at the sites of surrounding atoms, within a sphere of radius *r*_cut_. It is defined in three dimensions as
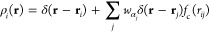
5where the sum is over all atoms within *r*_cut_ from the central atom. Here, **r**_*i*_ is the position of atom *i*, *r*_*ij*_ = |**r**_*i*_ – **r**_*j*_|, *w*_α_*j*__ is the specie-specific weight of atom *j*, and *f*_c_ is a cutoff function that smoothly runs to zero as *r*_*ij*_ approaches *r*_cut_, as defined in ref (25). In order to represent this density distribution as a vector, it is expanded on a suitable basis. Atomic positions are first mapped onto the 4D sphere, by switching to polar coordinates (θ,ϕ,*r*) and by defining a third polar angle, θ_0_, from the radial coordinate (see ref (23) for details). The density function is then expanded in terms of hyperspherical harmonics , the natural basis for expansion on the 4D sphere. Dropping the atomic index, ρ is written as
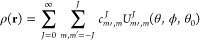
6

The hyperspherical harmonic index, *J*, runs in half-integer steps, while *m* and *m*′ run between −*J* and *J* in integer steps. The outer sum is truncated in practice at a value of *J*_max_, treated as a hyperparameter. The expansion coefficients, , cannot be used as descriptors since they are complex and are not invariant under system rotation. From them, however, the rotationally invariant and real-valued bispectrum components  are constructed
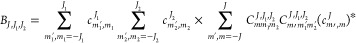
7

Here,  and  are the Clebsch–Gordan coefficients, which possess the same symmetry invariances as the system. After taking the nonzero and unique distinct components, the bispectrum vector is formed, denoted , with atomic and specie indices. The bispectrum components are a highly nonlinear representation of the local atomic coordinates and account for up to four-body interactions. Their complexity is what makes it possible for them to be effectively used together with a simple regressor in SNAP to accurately map structures to energies.

The fitting, testing, and predictions of the SNAP models used are performed using an in-house Python library built with SCIKIT-LEARN^76^ and the ASE^63^ Python libraries. The bispectrum components are computed using LAMMPS.^77^ The pipeline is built in Python to perform the API download of binary structures and energies from the AFLOWlib^78^ database and to generate derivative structures from the prototypes using ENUMLIB.^53^ DFT calculations are managed by using a combination of ASE^63^ and PYMATGEN.^79^

## Data Availability

Data associated with this project, including the AFLOWlib auid for the training data, the AFLOWlib labels of the structures used as prototypes, the parameters of the SNAP models, and the lowest values of the enthalpy of formation found at each composition are available on the Github repository (https://github.com/HugoRossignol/Workflow_Ternary_ConvexHull).

## Supporting Information Available

Information on the prototype structures: AFLOWlib auid, space group of the parent binary compound, and space group of the prototype structure for the Cu–Ag–Au and Mo–Ta–W systems (PDF)

## Footnotes

a By this, we mean all permutations of a stoichiometric ratio. For an *X*–*Y*–*Z* ternary system, 2–1–1 refers to three compositions: *X*_2_*Y*_1_*Z*_1_, *X*_1_*Y*_2_*Z*_1_, and *X*_1_*Y*_1_*Z*_2_.
