# Supplementary material for: Machine-Learning-Assisted Construction of Ternary Convex Hull Diagrams
Source: J Chem Inf Model. 2024 Jan 25;64(6):1828–40. doi: 10.1021/acs.jcim.3c01391 (PMC10966649; doi:10.1021/acs.jcim.3c01391)
Supplement: Supplementary file 1 — ci3c01391_si_001.pdf [file ci3c01391_si_001.pdf]

Machine-Learning-Assisted Construction of Ternary Convex Hull Diagrams

Hugo Rossignol[1], Michail Minotakis[1], Matteo Cobelli[1], and Stefano Sanvito[1]

[1]School of Physics and CRANN Institute, Trinity College Dublin, College Green, Dublin 2, Ireland

S1 Binary Prototypes

Table S1: AFLOWlib auid and space group of the binary compounds selected as prototypes to generate the candidate ternary compounds for the Cu-Ag-Au system. The final column is the space group of the structure of the prototype (all atomic sites are identical).

**AFLOWlib auid**	**Space Group of Binary**	**Space Group of Prototype**
aflow:13372e31f41c850a	225	225
aflow:68ce17d9a7e916e2	194	194
aflow:917f75ef47f43532	62	15
aflow:609f4adcc4fcca86	139	225
aflow:93dc810d8c30f10d	194	194
aflow:f7519e1bbb417c9c	221	225
aflow:1058ee2b8abcb7a6	139	166
aflow:8103c1b24e47a010	87	166
aflow:6f3f5b696f5aa391	123	139
aflow:0354ff34a2e8977d	129	12
aflow:38accf5c391aae61	139	139
aflow:745557da358b7599	164	12
aflow:b9cf690847e0d975	225	225
aflow:c2f26f7969564d16	166	166
aflow:6f3480c0529b7f44	65	12
aflow:6dd2daa624a1e063	141	2

Table S1: AFLOWlib auid and space group of the binary compounds selected as prototypes to generate the candidate ternary compounds for the Cu-Ag-Au system. The final column is the space group of the structure of the prototype (all atomic sites are identical).

**AFLOWlib auid**	**Space Group of Binary**	**Space Group of Prototype**
aflow:7e5e25d02ef3588b	65	12
aflow:2b272f63a4c9f7b9	229	229
aflow:93a31bc6678cd60d	71	12
aflow:6c62d8825020a470	12	12
aflow:8273e01e7a094dbf	8	8
aflow:a73395b2e1cd969a	38	38
aflow:69a8361cc1cb1112	123	123
aflow:87c1fae46433ffc9	194	194
aflow:635ad0651547e25e	227	227
aflow:3e58fc5ba92951bf	164	164
aflow:c162c3e2f4de9c79	139	139
aflow:dc47422a610dc3bf	194	194
aflow:7285868a88ccc369	123	123
aflow:a974fcb1e59b0a35	51	51
aflow:b554d99a1a47e8ed	63	63
aflow:fb5bb81572653498	139	139
aflow:3e06df2d581f2e68	59	59
aflow:22252eae86f6a1f4	194	194
aflow:6ed221d6611d7ca6	123	139
aflow:7472cdf89358d5e6	11	11
aflow:c81b8d71a097d6df	123	123
aflow:2be0bd6440d6bc77	12	71
aflow:5addbb8df6d6403f	139	139
aflow:dc5e8311267c00ee	65	69
aflow:1faed9c566dea1dc	69	69
aflow:d39fb2cdb102d201	71	71
aflow:88cdd0c9696f4d12	123	139
aflow:2c082f6dca0164b2	139	139
aflow:aab64cf2e25924b8	139	139

Table S2: AFLOWlib auid and space group of the binary compounds selected as prototypes to generate the candidate ternary compounds for the Mo-Ta-W system. The final column is the space group of the structure of the prototype (all atomic sites are identical).

**AFLOWlib auid**	**Space Group of Binary**	**Space Group of Prototype**
aflow:13f126f5d2da4daa	221	229
aflow:56b5774e29e763d7	12	139
aflow:0a9ded068f1b3fab	12	12
aflow:539264a32e8a18c7	139	139
aflow:d1f20383523c30e5	12	12
aflow:70c6dd99069de8f8	166	166
aflow:7ca9bed88b4117e9	12	12
aflow:a2241c8314d7a201	71	71
aflow:957658abcdb8b053	129	129
aflow:61e6c90baeb1c403	139	139
aflow:4d11371ad48b0021	123	123
aflow:88261d91222b7772	12	12
aflow:f696359d7fbe5f86	12	12
aflow:e1178814f8e2eb1b	12	229
aflow:48c7053ad9d2e524	63	139
aflow:826f41e062127275	63	229
aflow:a6492b22f2d3cf56	87	229
aflow:462101230eb0904b	164	229
aflow:fb86d1f1f7e9b3d4	8	69
aflow:b0bb4c5f5c36ed88	8	12
aflow:c54450136fc1b12a	74	74
aflow:237fd0238dcc4efc	71	71
aflow:512cfa160db51cc0	12	12
aflow:b0a24a01231b70a8	123	123
aflow:f6e18b7ffd617792	12	69
aflow:637d02a1a8b5fa0d	63	63
aflow:746ba9f8907b2a85	87	87
aflow:fb43fad2c5f66f16	221	229
